# Identification of optimal contemporary antiemetic prophylaxis for doxorubicin-cyclophosphamide chemotherapy in Chinese cancer patients: post-hoc analysis of 3 prospective studies

**DOI:** 10.20892/j.issn.2095-3941.2020.0241

**Published:** 2021-08-15

**Authors:** Winnie Yeo, Leung Li, Thomas KH Lau, Kwai T Lai, Vicky TC Chan, Kwan H Wong, Christopher CH Yip, Elizabeth Pang, Maggie Cheung, Vivian Chan, Carol CH Kwok, Joyce JS Suen, Frankie KF Mo

**Affiliations:** 1 Department of Clinical Oncology, Prince of Wales Hospital, Faculty of Medicine, Hong Kong Cancer Institute, Hong Kong, China; 2 State Key Laboratory of Translational Oncology, The Chinese University of Hong Kong, Hong Kong, China; 3 Department of Clinical Oncology, Princess Margaret Hospital, Hong Kong, China

**Keywords:** Netupitant, palonosetron, aprepitant, olanzapine, NEPA, Asians

## Abstract

**Objective:**

Chemotherapy-induced nausea and vomiting (CINV) are common with doxorubicin-cyclophosphamide (AC) chemotherapy. Recommended antiemetic regimens incorporate neurokinin-1 receptor antagonist (NK1RA), 5-hydroxytryptamine type-3 receptor antagonist (5HT3RA), corticosteroid, and dopamine antagonists. This post-hoc analysis compared results of 3 prospective antiemetic studies conducted among Chinese breast cancer patients who received (neo)adjuvant AC, in order to identify optimal antiemetic prophylaxis.

**Methods:**

A total of 304 patients were included: Group 1, ondansetron/dexamethasone (D1); Group 2, aprepitant/ondansetron/dexamethasone (D1); Group 3, aprepitant/ondansetron/dexamethasone (D1–3); Group 4, aprepitant/ondansetron/dexamethasone (D1–3)/olanzapine; and Group 5, netupitant/palonosetron/dexamethasone (D1–3). Antiemetic efficacies of Groups 3, 4, and 5 during cycle 1 of AC were individually compared with Group 1. In addition, emesis outcomes of patients in Groups 3 and 5, and those of Groups 2 and 3, were compared.

**Results:**

When comparing efficacies of a historical doublet (5HT3RA/dexamethasone) with triplet antiemetic regimens (NK1RA/5HT3RA/dexamethasone) with/without olanzapine, complete response (CR) percentages and quality of life (QOL) in overall phase of cycle 1 AC were compared between Group 1 and the other groups: Group 1 *vs.* 3, 41.9% *vs.* 38.3% (*P* = 0.6849); Group 1 *vs.* 4, 41.9% *vs.* 65.0% (*P* = 0.0107); and Group 1 *vs.* 5, 41.9% *vs.* 60.0% (*P* = 0.0460). Groups 4 and 5 achieved a better QOL. When comparing netupitant-based (Group 3) with aprepitant-based (Group 5) triplet antiemetics, CR percentages were 38.3% *vs.* 60.0%, respectively (*P* = 0.0176); Group 5 achieved a better QOL. When comparing 1 day (Group 2) *vs.* 3 day (Group 3) dexamethasone, CR percentages were 46.8% and 38.3%, respectively (*P* = 0.3459); Group 3 had a worse QOL.

**Conclusions:**

Aprepitant-containing triplets were non-superior to doublet antiemetics. Netupitant-containing triplets and adding olanzapine to aprepitant-containing triplets were superior to doublets. Netupitant/palonosetron/dexamethasone was superior to aprepitant/ondansetron/dexamethasone. Protracted administration of dexamethasone provided limited additional benefit.

## Introduction

Cytotoxic chemotherapy is an essential component of adjuvant therapy for patients with early stage as well as locally advanced breast cancer. The combination of doxorubicin and cyclophosphamide, commonly known as AC, remains one of the most frequently adopted neo/adjuvant chemotherapeutic regimens. The AC (or AC-like) regimen has been regarded as highly emetogenic. Chemotherapy-induced nausea and vomiting (CINV) are highly distressing symptoms; ineffective control of CINV can adversely affect quality of life, which in turn may lead to poor treatment compliance and affect an individual’s prognosis^1–3^. Prophylactic antiemetic treatment paradigms until the mid-2000s were based on doublets of the first generation 5-hydroxytryptamine type-3 receptor antagonist (5HT3RA) and corticosteroids. However, contemporary international guidelines from the European Society of Medical Oncology/Multinational Association Supportive Care in Cancer (ESMO/MASCC), the American Society of Clinical Oncology (ASCO), and the US National Comprehensive Cancer Network (NCCN), recommended antiemetic prophylaxis include a combination of 5-HT3RA and corticosteroids with aneurokinin-1 receptor antagonist (NK1RA) and/or olanzapine^4–6^. While the recommended antiemetic regimens for the control of acute CINV within the first 24 h of AC are similar, recommendations for preventing delayed CINV (from 24–120 h after chemotherapy) differ slightly between guidelines in terms of duration of dexamethasone and use of olanzapine in relation to specific antiemetic combinations.

In this report, we conducted post-hoc analysis of individual patient data gathered from 3 previously reported studies that tested efficacies of 5 different antiemetic regimens to determine the optimal antiemetic regimen for Chinese patients with breast cancer^7–9^.

## Materials and methods

Three prospective studies were included in the present analyses. Study A was a randomized placebo controlled study completed in 2007 that assessed the control of CINV using ondansetron/dexamethasone with or without aprepitant^7^ Study B was a randomized study conducted between 2017 and 2018 that assessed aprepitant/ondansetron/dexamethasone triplet regimen with or without olanzapine^8^; and Study C was a prospective single arm study conducted in 2018–2019 that assessed the antiemetic efficacy of netupitant/palonosetron/dexamethasone^9^.

Eligibility criteria of all 3 studies were similar and included female Chinese patients aged > 18 years with early stage breast cancer, chemotherapy-naïve, planned for (neo)adjuvant AC, and being able to read, understand, and complete the study diary and questionnaires in Chinese. Similar exclusion criteria were used in all studies^7–9^. The studies were approved by the Joint CUHK-NTEC Institution Review Board of the Chinese University of Hong Kong and of the Hong Kong Hospital Authority, and the Kowloon West Cluster Research Ethics Committee of the Hong Kong Hospital Authority [Approval Nos. CREC-2002-321, CREC-2016.013, CREC-2017.169 and KW/FR-18-019 (119-19)]. Studies B and C were registered at ClinicalTrials.gov (Identifier: NCT03386617 and NCT03079219, respectively). Consent was obtained from all patients.

### Study treatment

For the purpose of the present analyses, the antiemetic regimens tested in the 3 studies were categorized into 5 groups, details of which are listed in **Table 1**. Of note, Group 1, which consisted of only ondansetron and dexamethasone, would commonly be regarded as a historical antiemetic regimen by contemporary standards. Patients were instructed to take rescue therapy if needed for nausea or vomiting as stipulated in the respective study protocol^7–9^.

**Table 1:** Treatment arms of the 3 prospective studies of Chinese breast cancer patients undergoing doxorubicin-cyclophosphamide chemotherapy

Studies	Treatment arms	Patient No.	Day 1	Day 2	Day 3	Day 4	Day 5
Study A*	Group 1	62	Ondansetron 8 mg twice + Dexamethasone 20 mg	Ondansetron 8 mg twice	Ondansetron 8 mg twice	–	–
	Group 2	62	Aprepitant 125 mg + Ondansetron 8 mg twice + Dexamethasone 12 mg	Aprepitant 80 mg	Aprepitant 80 mg	–	–
Study B	Group 3	60	Aprepitant 125 mg + Ondansetron 8 mg twice + Dexamethasone 12 mg	Aprepitant 80 mg + Dexamethasone 8 mg	Aprepitant 80 mg + Dexamethasone 8 mg	–	–
	Group 4	60	Aprepitant 125 mg + Ondansetron 8 mg twice + Dexamethasone 12 mg + olanzapine 10 mg	Aprepitant 80 mg + olanzapine 10 mg	Aprepitant 80 mg + olanzapine 10 mg	Olanzapine 10 mg	Olanzapine 10 mg
Study C	Group 5	60	Netupitant 300 mg** + Palonosetron 0.5 mg** + Dexamethasone 12 mg	Dexamethasone 8 mg	Dexamethasone 8 mg	–	–

*Placebo controlled study.

**These were combined in a capsule, NEPA.

### Assessment of antiemetic efficacies

All 3 studies had similar study procedures. AC chemotherapy was given on day 1 (D1). Within the first 120 h of AC infusion, each patient recorded the date and time of any vomiting episodes and the use of rescue medication. On D2–6, symptoms of nausea for the preceding 24 h were rated using a visual analogue scale (VAS), where 0 mm implied no nausea and 100 mm implied nausea that was “as bad as it could be.” On D6, individuals completed the Chinese version of the Functional Living Index-Emesis (FLIE) questionnaire. A research team contacted individual patients during D2–6 regarding the study procedures. Treatment compliance was based on records of time, date, and number of tablets taken each day.

Assessment started from the initiation of AC (0 h) to 120 h after chemotherapy infusion over 3 time frames: “acute” phase referred to 0–24 h after initiation of AC, “delayed” phase referred to 24–120 h, while “overall” phase referred to 0–120 h. The variables used to measure antiemetic efficacy were as follows: the proportion of patients with “complete response” (CR; defined as no vomiting and no use of rescue therapy), the proportion of patients reporting “no vomiting” (NV; no vomiting or retching including patients who received rescue therapy), “no significant nausea” (NSN; nausea VAS < 25 mm), and “no nausea” (NN; nausea VAS < 5 mm). These assessments were done primarily over the overall phase, but were also conducted separately during acute and delayed phases.

Quality of life (QOL) analysis using FLIE was evaluated as previously reported^4–6^; 3 parameters were measured: nausea domain, vomiting domain, and total score, where calculated and lower scores reflected a better QOL^10^.

### Statistical analysis

The primary objective was to identify the optimal antiemetic regimen by comparing the efficacies of individual antiemetic regimens with or without an NK1RA during cycle 1 AC. Secondary objectives were to compare the antiemetic efficacy of (1) netupitant-based *vs.* aprepitant-based triplet antiemetic regimens; and (2) 1-day *vs.* 3-day dexamethasone.

The modified intention-to-treat approach was used for all efficacy analyses. Only patients who had received chemotherapy and had completed 120 h of study procedures in cycle 1 AC were included in the analysis.

To address the primary objective of this study, antiemetic efficacies of Groups 3, 4, and 5 during cycle 1 of AC were individually compared with Group 1. The percentages of patients who achieved CR during the acute, delayed, and overall phases post-chemotherapy infusion were compared. Other parameters including NV, NSN, and SN percentages as well as QOL were compared.

To compare the efficacy of netupitant-based *vs.* aprepitant- based triplet antiemetic regimens, emesis outcomes of patients in Groups 3 and 5 were assessed. To assess the antiemetic efficacy of 1-day *vs.* 3-day dexamethasone, emesis outcomes of patients in Groups 2 and 3 were compared. The main parameter that was compared was CR in cycle 1. Other parameters that were compared in cycle 1 were NV, NSN, NN, and QOL. In addition, assessment over multiple cycles was conducted to compare the percentages of CR in the acute, delayed, and overall time frames.

Comparisons between 2 arms were made using the Wilcoxon rank sum test for continuous data and the chi-square test for dichotomous data with a 2-sided significance level of 5%.

## Results

A total of 304 patients participated in the 3 studies. Details of background characteristics of the study populations have been described in earlier reports^4–6^. Patients were similar in most characteristics apart from age (where Groups 1 and 2 patients appeared slightly younger at medians of 48.0 and 46.5 years, respectively, while patients of Groups 3, 4, and 5 were 55.5, 54.5, and 56 years, respectively) and treatment setting (where 25% of patients in Groups 3, 4, and 5 received AC as neoadjuvant therapy but none in Groups 1 and 2 received AC).

### Comparison of antiemetic efficacies in regimens with or without an NK1RA during cycle 1 of AC

**Table 2** shows the emesis outcomes of Groups 3, 4, and 5 compared with those of Group 1; the previously reported outcomes between Groups 1 and 2 were included as a reference^4^.

**Table 2:** Antiemetic efficacies during cycle 1 of the AC emesis endpoint of Groups 3, 4, and 5 individually compared with Group 1

	Group 1	Group 2*		Group 3		Group 4		Group 5	
	Outcomes	Outcomes	*P*	Outcomes	*P*	Outcomes	*P*	Outcomes	*P*
Acute (0–24 h), *n* (%)									
NV	46 (74.2)	44 (72.1)	0.7963	31 (51.7)	0.0099	44 (73.3)	0.9140	43 (71.7)	0.7534
NSN	52 (83.9)	54 (88.5)	0.4546	45 (75.0)	0.2249	57 (95.0)	0.0464	52 (86.7)	0.6633
NN	37 (59.7)	38 (62.3)	0.7660	32 (53.3)	0.4797	46 (76.7)	0.0443	42 (70.0)	0.2328
CR	45 (72.6)	44 (72.1)	0.9556	31 (51.7)	0.0172	42 (70.0)	0.7527	42 (70.0)	0.7527
Delayed (24–120 h), *n* (%)									
NV	31 (67.4)	34 (75.6)	0.3887	24 (77.4)	0.3394	41 (93.2)	0.0018	37 (86.0)	0.0383
NSN	39 (75.0)	40 (74.1)	0.9129	38 (84.4)	0.2515	55 (96.5)	0.0010	47 (90.4)	0.0381
NN	22 (59.5)	18 (47.3)	0.2940	20 (62.5)	0.7963	35 (76.1)	0.1045	32 (76.2)	0.1106
CR	26 (57.8)	29 (64.4)	0.5165	23 (74.2)	0.1417	39 (92.9)	0.0001	36 (85.7)	0.0040
Overall time frame (0–120 h), *n* (%)									
NV	31 (50.0)	34 (54.8)	0.5896	24 (40.0)	0.2671	41 (68.3)	0.0395	37 (61.7)	0.1946
NSN	39 (62.9)	41 (66.1)	0.7074	38 (63.3)	0.9607	55 (91.7)	0.0002	47 (78.3)	0.0617
NN	22 (35.5)	19 (30.6)	0.5669	20 (33.3)	0.8026	35 (58.3)	0.0114	32 (53.3)	0.0472
CR	26 (41.9)	29 (46.8)	0.5876	23 (38.3)	0.6849	39 (65.0)	0.0107	36 (60.0)	0.0460
FLIE scores, mean (standard deviation)**									
Nausea domain	32.46 (32.32)	27.44 (25.70)	0.8059	27.71 (28.33)	0.5246	8.39 (17.02)	<0.0001	17.55 (28.03)	0.0040
Vomiting domain	24.03 (30.77)	3.49 (13.14)	<0.0001	10.69 (19.99)	0.1280	3.63 (11.45)	0.0045	6.74 (22.40)	0.0016
Total score	28.2 (30.48)	15.5 (16.03)	0.2509	19.2 (20.78)	0.3116	6.01 (13.31)	<0.0001	12.14 (23.26)	0.0012

*Comparisons between Group 1 and 2 were previously reported^4^.

**Scorings of each item ranged from a 1 to 7 point scale that was based on a 100 mm visual analogue scale. For most items, the higher the score, the worse the impact was on the patient’s quality of life; for some items, the opposite stands and these scores were transformed back to having the same direction as the majority of the items.

NV, no vomiting; NSN, no significant nausea; NN, no nausea; CR, complete response.

When Group 1 [ondansetron (D1–3)/dexamethasone (D1)] was compared with Group 3 [aprepitant (D1–3)/ondansetron (D1)/dexamethasone (D1–3)], the CR percentage was significantly higher in Group 1 during the acute, but not the delayed and overall, phases. Group 1 also had significantly higher percentages of NV in the acute phase.

When Group 1 was compared with Group 4 [aprepitant (D1–3)/ondansetron (D1)/dexamethasone (D1)/olanzapine (D1–5)], CR percentages were significantly higher in Group 4 for the delayed (57.8% *vs.* 92.9%, *P* = 0.0001) and overall (41.9% *vs.* 65.0%, *P* = 0.0107) phases. Other parameters were also significantly improved in Group 4, including NN in the acute and overall phases, NV in the delayed and overall phases, and NSN in all 3 phases.

Finally, when Group 1 was compared with Group 5 [netupitant (D1)/palonosetron (D1)/dexamethasone (D1–3)], CR percentages were significantly higher in Group 5 for the delayed (57.8% *vs.* 85.7%, *P* = 0.0040) and overall (41.9% *vs.* 60.0%, *P* = 0.0460) phases. Other parameters, including NV and NSN in the delayed phase, and NN in the overall phase, were also significantly better in Group 5.

When compared to Group 1, no difference in QOL was observed with Group 3, while significantly better QOLs in all 3 parameters of FLIE were detected in both Group 4 and Group 5 (**Table 2**).

### Comparison of antiemetic efficacies of netupitant-based and aprepitant-based triplet antiemetic regimens

Outcomes of patients in Group 3 [netupitant (D1)/palonosetron (D1)/dexamethasone (D1–3)] and Group 5 [aprepitant (D1–3)/ondansetron (D1)/dexamethasone (D1–3)] were assessed. During cycle 1 AC, CR percentages were significantly higher in Group 5 during the acute (51.7% *vs.* 70.0%, *P* = 0.0397) and overall (38.3% *vs*. 60.0, *P* = 0.0176) phases. Other parameters that were also significantly better in Group 5 included NV in the acute phase (51.7% *vs.* 71.7%, *P* = 0.0243) as well as NV (40.0% *vs.* 61.7%, *P* = 0.0176) and NN (33.3% *vs.* 53.3%, *P* = 0.0271) in the overall phase. Group 5 patients experienced significantly better QOLs for all 3 FLIE parameters. Specifically, for Group 3 *vs.* Group 5, mean (standard deviation) FLIE scores for the nausea domain were 27.71 (28.33) *vs.* 17.55 (28.03) (*P* = 0.0114) and those for the vomiting domain were 10.69 (19.99) *vs.* 6.74 (22.40) (*P* = 0.0169), while the corresponding values for total score were 19.2 (20.78) *vs.* 12.14 (23.26) (*P* = 0.0037).

During multiple cycle assessment, Group 5 patients had higher percentages of CR across all 4 AC cycles (**Table 3A**); with the exception of those assessed in the delayed phase of cycles 1 and 2, with the findings were statistically significant across all phases of the 4 cycles.

**Table 3A:** Comparison of antiemetic efficacy in terms of complete response over multiple cycles: Group 3 *vs*. Group 5

	Acute (0–24 h), %			Delay (24–120 h), %			Overall (0–120 h), %		
	Group 3 *n* (%)	Group 5 *n* (%)	*P*	Group 3 *n* (%)	Group 5 *n* (%)	*P*	Group 3 *n* (%)	Group 5 *n* (%)	*P*
Cycle 1	31 (51.7)	42 (70.0)	0.0397	23 (74.2)	36 (85.7)	0.2165	23 (38.3)	36 (60.0)	0.0176
Cycle 2	39 (66.1)	51 (85.0)	0.0164	34 (87.2)	47 (92.2)	0.4354	34 (57.6)	47 (78.3)	0.0154
Cycle 3	39 (66.1)	53 (88.3)	0.0038	34 (87.2)	52 (98.1)	0.0428	34 (57.6)	52 (86.7)	0.0004
Cycle 4	42 (71.2)	53 (88.3)	0.0198	34 (81.0)	52 (98.1)	0.0053	34 (57.6)	52 (86.7)	0.0004

### Comparison of CINV with 1-day vs. 3-day dexamethasone

Emesis outcomes of patients in Group 2 [aprepitant (D1–3)/ondansetron (D1)/dexamethasone (D1)] and Group 3 [aprepitant (D1–3)/ondansetron (D1)/dexamethasone (D1–3)] were compared.

During cycle 1 of AC, there were significantly higher percentages of CR in Group 2 during the acute phase (67.2% *vs.* 51.7%, *P* = 0.0204). The percentage of patients with NV during the acute phase was also significantly higher in Group 2 (72.1% *vs.* 51.7%, *P* = 0.0204). QOL assessment revealed that although Group 2 patients had significantly better QOL in the vomiting domain of FLIE, the mean (standard deviation) scores were 3.49 (13.14) *vs.* 10.69 (19.99) (*P* = 0.0002); there was no difference detected in the nausea domain or total score.

During multiple cycle assessment, Group 2 had significantly higher CR percentages in the acute phase throughout the 4 AC cycles (**Table 3B**). However, in the delayed phase, Group 3 had significantly higher CR percentages during cycles 2 and 3, while there were no differences detected in the overall phase.

**Table 3B:** Comparison of antiemetic efficacies in terms of complete response over multiple cycles: Group 2* vs*. Group 3

	Acute (0–24 h), %			Delay (24–120 h), %			Overall (0–120 h), %		
	Group 2 *n* (%)	Group 3 *n* (%)	*P*	Group 2 *n* (%)	Group 3 *n* (%)	*P*	Group 2 *n* (%)	Group 3 *n* (%)	*P*
Cycle 1	44 (72.1)	31 (51.7)	0.0204	29 (64.4)	23 (74.2)	0.3689	29 (46.8)	23 (38.3)	0.3459
Cycle 2	55 (91.7)	39 (66.1)	0.0006	36 (65.5)	34 (87.2)	0.0173	40 (66.7)	34 (57.6)	0.3093
Cycle 3	54 (90.0)	39 (66.1)	0.0016	37 (68.5)	34 (87.2)	0.0367	41 (68.3)	34 (57.6)	0.2264
Cycle 4	53 (89.8)	42 (71.2)	0.0106	35 (66.0)	34 (81.0)	0.1054	42 (71.2)	34 (57.6)	0.1240

## Discussion

Despite adopting identical antiemetic regimens that were tested in a large international study among patients planning for AC-like chemotherapy^11^, our earlier study reported that the addition of aprepitant did not improve emesis control over a doublet of ondansetron/dexamethasone, a combination that is now regarded as suboptimal^4^. Using available data from 3 previously reported prospective studies that consisted of a homogenous breast cancer patient population of Chinese ethnicity who were planned for 4 cycles of AC (neo)adjuvant chemotherapy, the current post-hoc analysis facilitated comparisons of efficacies between historical doublet *vs.* triplet (NK1RA-containing) antiemetic regimens with/without olanzapine. The findings revealed that there was no advantage with prolonging dexamethasone administration to 3 days (Group 3) when administering an aprepitant-containing triplet antiemetic regimen. This finding could impact significantly on the choice of antiemetic agents to be used, especially in patients who have peptic disease or diabetics, where the lowest effective dose of dexamethasone should be considered. However, by adding olanzapine to aprepitant/ondansetron/dexamethasone (Group 4), control of CINV was significantly improved in several aspects, including better QOL; as such, the use of olanzapine is encouraged, especially taking into consideration its low cost. However, the advantage of adding an NK1RA to a doublet regimen was only evident when the netupitant/palonosetron/dexamethasone triplet regimen was used (Group 5), and this was associated with improved overall QOL. Our findings are consistent with the current international recommendations on the use of olanzapine. Notably, a recent large-scale randomized study comparing olanzapine with placebo in the presence of a triple antiemetic regimen provides further support for the use of olanzapine at an even lower daily dose of 5 mg^12^.

Few studies have directly compared the efficacy of aprepitant-containing *vs*. netupitant-containing triplet antiemetic regimens. In an earlier study that involved 413 patients undergoing moderately or highly emetogenic chemotherapy, although patients randomized to netupitant/palonosetron/dexamethasone consistently showed a small numerical advantage of 2%–7% in CR percentages across multiple cycles, the differences were not significantly different from those randomized to aprepitant/palonosetron/dexamethasone^13^. Subsequently, Zhang et al.^14^ conducted a randomized study in 800 Asian patients who were receiving a cisplatin-containing regimen, when compared to the aprepitant/granisetron/dexamethasone arm, the only significant finding was a lower requirement of rescue therapy in the netupitant/palonosetron/dexamethasone arm. In our recent report on breast cancer patients undergoing AC, netupitant/palonosetron/dexamethasone was shown to be superior to historical controls treated with aprepitant/ondansetron/dexamethasone^6^. However, the findings were postulated to be attributed by the fact that netupitant-treated patients received 3 days while the historical controls only had 1 day of dexamethasone. To eliminate the potential effect of protracted dexamethasone, the current analysis compared emesis outcomes of patients treated with aprepitant/ondansetron (Group 3) *vs.* netupitant/palonosetron (Group 5) in the presence of the same duration of dexamethasone for 3 days. The results revealed that during cycle 1 AC, CR percentages were significantly higher in Group 5 during the acute phase and overall time frame. In addition, several other emesis endpoints were also significantly better and these were associated with improved overall QOL. This observation confirmed preclinical findings that netupitant had longer acting efficacy than other NK1RAs^15,16^. In addition, while palonosetron, a second generation 5HT3RA, has been shown to be more potent than the first-generation counterparts, laboratory investigation has also shown that palonosetron significantly inhibits the substance P-mediated response and synergistically enhances the effect of netupitant^17–19^.

Although the recommendations for the use of antiemetics during the acute phase are similar in various guidelines, details for the delayed phase vary slightly. Specifically, ASCO has not included dexamethasone beyond day 1 of AC^1^. However, while ESMO/MASCC has similar recommendations when NEPA is used as the NK1RA/5HT3RA partner, it recommends the extension of dexamethasone over days 2–3 as an alternative to aprepitant over the same period^2^. Conversely, irrespective of whether NEPA or aprepitant is used, NCCN suggests the option of including dexamethasone for 4 days^3^. While Roscoe et al.^20^ has reported that more protracted use of dexamethasone after chemotherapy decreases delayed nausea; similar observations were not reported in another study^21^. Although dexamethasone in combination with other antiemetic agents is recognized to be safe^22^, it has been shown to cause moderate-to-severe adverse effects, such as insomnia, gastrointestinal symptoms, agitation, increased appetite, and weight gain^23^. Besides, dexamethasone could potentially worsen diabetic control, aggravate osteopenia/osteoporosis, and cause deterioration in cataracts. Accordingly, minimizing the total dose of prophylactic dexamethasone in patients undergoing multiple cycles of emetogenic chemotherapy could be advantageous.

In view of the above, we have pooled data from Groups 2 and 3. These patients were given the same doses of aprepitant/ondansetron but different durations of dexamethasone, i.e., 1 day (Group 2) *vs.* 3 days (Group 3). Our results revealed that apart from higher CR percentages in the delayed phases of cycles 2 and 3, there was limited additional benefit with prolonged administration of dexamethasone. Our findings were consistent with an earlier randomized phase II study on 80 breast cancer patients receiving an AC-like regimen; using a triplet antiemetic regimen of palonosetron/aprepitant/dexamethasone, no significant improvement in emesis outcome was detected with 3 days of dexamethasone^24^. In addition, in a phase III trial where patients undergoing highly emetogenic chemotherapy were given an NK1RA (aprepitant or fosaprepitant) and palonosetron and randomized to 1-day or 3-day dexamethasone, non-inferiority in CR percentages for the overall time frame was reported^25^. The current results are further supported by a recent meta-analysis that included 5 studies testing palonosetron and dexamethasone on patients undertaking AC-based or non-AC moderately emetogenic chemotherapy^26^.

Findings from the current report are limited by a number of factors. First, the 3 studies presented in this analysis were not conducted concurrently; rather, the findings were based on 3 separate studies, each analyzed separately, with a span of approximately 10 years between the first study and the other more recent studies. Despite the use of identical antiemetic agents on day 1, there were remarkable percentages for the control of CINV in Group 2, which were significantly higher than that of Group 3 during the acute phases across the 4 AC cycles. As such, cross-study comparisons should be interpreted with caution. An additional limitation was that only 1 of the 3 prospective studies was placebo-controlled; another one was an open-labeled randomized study, and the third was a single arm study. Nonetheless, the current report is strengthened by the fact that all 3 studies enrolled a uniform patient population, namely, breast cancer patients of Chinese ethnicity who had early stage disease and were all treated with AC.

## Conclusions

Based on the current analyses of breast cancer patients who received AC, an aprepitant-containing triplet regimen was not superior to a historical doublet regimen of ondansetron/dexamethasone, while the netupitant-containing triplet and the addition of olanzapine to aprepitant-containing triplet regimens were individually superior to the doublet regimen. Netupitant/palonosetron/dexamethasone was superior to the aprepitant/ondansetron/dexamethasone triplet antiemetic regimen. Furthermore, protracted administration of dexamethasone provided limited additional benefit for the control of CINV. Our findings are consistent with that of a recent meta-analysis, in which olanzapine-containing regimens were indicated to be the most effective in controlling CINV, while a netupitant/palonosetron-containing regimen was more effective than other conventional NK1RA-containing triplet regimens^27^. The applicability of the current findings to other patient populations receiving highly emetogenic chemotherapeutic regimens other than AC remains to be confirmed.
